# Sympathomimetic effects of chronic methamphetamine abuse on oral health: a cross-sectional study

**DOI:** 10.1186/s12903-016-0218-8

**Published:** 2016-05-26

**Authors:** Niklas Rommel, Nils H. Rohleder, Steffen Koerdt, Stefan Wagenpfeil, Roland Härtel-Petri, Klaus-Dietrich Wolff, Marco R. Kesting

**Affiliations:** Department of Oral and Maxillofacial Surgery, Klinikum rechts der Isar, Technische Universität München, Ismaninger Str. 22, Munich, D-81675 Germany; Institute for Medical Biometry, Epidemiology and Medical Informatics, Saarland University, Campus Homburg, Kirrberger Straße 100, Homburg, D-66424 Germany; Department of Addiction Medicine, Hospital for Psychiatry, Psychotherapy, Psychosomatic Medicine, and Neurology, Nordring 2, D-954, 44 Bayreuth, Germany

**Keywords:** Methamphetamine, Saliva, Bruxism, Trismus, Matched-pair analysis, Cross-sectional study

## Abstract

**Background:**

Methamphetamine, a highly addictive sympathomimetic stimulant, is currently widely abused worldwide and has been associated with devastating effects on oral health, resulting in the term “meth mouth”. However, “meth mouth” pathology is primarily based on case reports with a lack of systematic clinical evaluation. Therefore, we have conducted a systematic study to investigate (1) the pharmacological impact of methamphetamine on oral health with regard to saliva function, including the parameters saliva flow rate and total saliva production (ml/5 min) and the buffering capacity of saliva; (2) the contribution of the symptoms of bruxism and muscle trismus to potential oral health damage.

**Methods:**

We assessed the data of 100 chronic methamphetamine abusers and 100 matched-pair comparison participants. Primarily, we conducted an anamnesis with all methamphetamine abusers with regard to saliva dysfunctions, jaw clenching and pain in the temporomandibular joint. Subsequently, in the first part of the clinical enquiry, we tested the saliva flow rate and the total saliva production (ml/5 min) by using the sialometry method and the buffer capacity of saliva by determining the pH-value. In the second part of the clinical enquiry, we evaluated bruxism symptoms with respect to generalized tooth attrition, dentine exposure and visible enamel cracks and examined a potential muscle trismus by measuring the maximal opening of the mouth.

**Results:**

The majority of methamphetamine abusers reported a dry mouth (72 %) and jaw clenching (68 %). Almost half of all methamphetamine abusers experienced pain in the temporomandibular joint (47 %). With regard to the clinical findings, methamphetamine abusers showed significantly lower total saliva production (ml/5 min) (*p* < 0.001), lower pH-values of their saliva (*p* < 0.001) and more bruxism symptoms (*p* < 0.001). However, we found no relevant trismus symptoms on comparing the two groups (*p* > 0.05).

**Conclusions:**

The sympathomimetic effects of chronic methamphetamine abuse may lead to dry mouth and extensive bruxism and therefore can increase the risk for caries decay, periodontal lesions and tooth wear. Furthermore, a significant decline of saliva buffer capacity in methamphetamine abusers may trigger the risk for dental erosions. Methamphetamine abusers and practitioners should be aware of these symptoms.

## Background

Methamphetamine (MA) is a highly addictive stimulant substance with deep historical roots. Originally, MA was synthesized by Nagayoshi Nagai in Japan in 1893 [1]. During the Second World War, the substance received political and military interest because of its disinhibiting effect and, above all, because it increased vigilance. At that time, the enormous potential of addiction to this substance became evident. Consumption of MA causes euphoria, subjectively stimulates performance and increases a person’s sense of self-esteem. The need for sleep and for assuaging hunger and thirst is reduced, whereas sexual desire and the flow of words are increased [1, 2]. These psychic and physical effects are brought about by the stimulation of receptors of noradrenalin and, in particular, of dopamine. MA prevents the reabsorption of dopamine into the synaptic vesicles and prolongs the effects of the substance in the synaptic cleft [1].

Currently, the use of MA is rapidly spreading and MA is widely abused with approximately 35 million MA abusers around the globe [1]. In many parts of the United States, MA seems to be replacing marijuana and crack cocaine as “the drug of choice” [3]. In Europe, MA is quickly expanding under the scene name of “Crystal” or “Crystal Meth” (CM), particularly in Germany and the Czech Republic. In a German Police Crime Statistic of 2013, a CM rise of almost 200 % has been noted in border areas to the Czech Republic during a two-year comparison from 2010 to 2012. Simultaneously, in the UK, the current abuse of CM is strongly increasing, particularly in London’s Gay Scene [4]. Individuals who chronically use CM/MA run a high risk of experiencing health hazards. However, MA use has particularly been associated with a severe impact on oral health [5–9]. The sociological conditions of the US health-care system led to the term “meth mouth” being coined in the medical literature for the first time in 2005 [10]. The pathogenic impact on oral health seems to be a pharmacological effect of methamphetamine. The substance is a central nervous stimulant and a strong activator of the sympathetic nervous system [1, 6]. These pharmacological effects can result in a dry mouth or even xerostomia [11–13] with a subsequent higher risk for carious lesions [14, 15], extensive grinding of the teeth [8, 10] or jaw clenching and muscle trismus [9, 10, 16]. However, “meth mouth’ symptoms and pathogenic mechanisms found in the literature are primarily based on assumptions and individual case reports, with a lack of conclusive relationships between chronic methamphetamine abuse and the aforementioned symptoms. Systematic study designs in this field are rare, mostly because of problematic access to a suitable number of MA abusers. Therefore, we have established a cooperation between addiction and maxillofacial medicine in order to conduct a systematic study comprising a large number of MA abusers. We consider that the sympathomimetic effects of the drug have a high impact on oral health. Hence, the aim of this study has been to examine (1) the pharmacological impact of MA on oral health with regard to saliva function, including the parameters saliva flow rate and total saliva production (ml/5 min) and the buffering capacity of saliva; (2) the contribution of the symptoms of bruxism and muscle trismus to potential oral health damage.

## Methods

### Study setting

This cross-sectional study design involved cooperation between two specialist clinics for addiction medicine in Upper Franconia, Germany, the Department of Oral and Maxillofacial Surgery of the Munich University of Technology, Germany and the Institute for Medical Biometry, Epidemiology and Medical Informatics of the University of Saarland, Germany. With the close consultation of each participating institution, the examining scheme, the number of participants and an information document setting out the objectives, risks and benefits of the study were established in advance of the start of the study. For the optimal comparability of data and the verification of our clinical examination methods, we recruited a comparison group including one same-aged and same-gender participant for each MA abuser. In this type of statistical comparison, defined as ‘matched-pair analysis’, a study group and a comparison group are made comparable with respect to extraneous factors by pairing study participants and comparison group participants individually based on matched-pair criteria. By matching study participants with a characteristic feature (in our case chronic MA abuse) to similar participants without this feature, matching enables a comparison of outcomes among these two groups in order to estimate the effect of the characteristic feature and to reduce bias due to confounding. In order to provide the study with adequate statistical power, a sample size calculation was performed for one of the main parameters of this study, namely ‘total saliva production (ml/5 min)’ (Table 2, Fig. 2), in consultation with the Institute for Medical Biometry, Epidemiology and Medical Informatics of Saarland University, as follows: a sample size of 100 in each group will have 80 % power to detect a difference in means of −1.0 (the difference between a Group 1 mean, μ_1_, of 3.0 and a Group 2 mean, μ_2_, of 4.0) assuming that the common standard deviation is 2.5 when a two group t-test is used with a 0.05 two-sided significance level. Therefore, the number of MA abusers and of the comparison group participants was set at 100. All study participants were provided with an information document setting out the objectives, risks and benefits of the study. According to the requirements of the ethics committee, we informed each participant about data use and data protection and obtained his or her written consent with regard to participation in the study. Our methods were approved by the local ethics committee of the Munich University of Technology (no. 5405/12) and were in accordance with the 1964 Helsinki declaration and its later amendments or comparable ethical standards.

### Participants

The selection and data acquisition of the 100 MA abusers took place at the addiction clinics between April 2012 and October 2013. In the addiction clinics, an average of 30 MA abusers underwent clinical withdrawal therapy for an average of 4 months with periodically changing patients. Primarily, the executive psychotherapist of each addiction clinic (2 psychotherapist overall) performed a short patient interview with each MA abuser in the clinic. The short patient interview included an examination of the inclusion and exclusion criteria and an explanation of the objectives, risks and benefits of the study. First, the inclusion criteria were checked. Inclusion criteria for the MA group included the constant MA abuse of 1 g/week beyond a minimum period of 12 months without any withdrawal periods (Table 1). This definition of chronic MA abuse was determined in consultation with the executive psychotherapists in the addiction clinics based on their personal and long-term experiences with chronic MA abusers and their addictive behavior. If the inclusion criteria were fulfilled, the exclusion criteria were checked. Exclusion criteria were defined as the last MA abuse of longer than 1 month previously and the excessive co-consumption of other addictive substances to a similar extent to MA (Table 1). If the MA abuser fulfilled the eligibility criteria, he or she was provided with an information document setting out the objectives, risks and benefits of the study, the executive psychotherapist being available in case of queries. Afterwards, each MA abuser was able to decide independently whether he or she wanted to join the study. 92 % of all questioned MA abusers agreed to participate in the study. Subsequently, at fixed examination appointments over 7-week periods on average, the clinical examination of all participants, including a short clinical anamnesis beforehand, was performed by a dentist and a maxillofacial surgeon in a prepared separate room guaranteeing a quiet atmosphere. The necessary examination equipment was provided by the Department of Oral and Maxillofacial Surgery of the Munich University of Technology and brought to each examination appointment.

**Table 1:** Participant data

	MA group (*n* = 100)	Comparison group (*n* = 100)
Inclusion criteria	- Constant MA use over a minimum period of 1 year - Constant MA use of 1 g/week by intranasal use, smoking, intravenous use or swallowing	- Same gender as MA user - Same age as MA user with a tolerance of ±1 year
Exclusion criteria	- Last MA use longer than 1 month ago - <18^th^ year of birth - Excessive co-consumption of other addictive substances to a similar extent to MA	-Any use of addictive substances in life
Recruitment and location of examination	- Addiction Clinic in Hochstadt/Main, Germany (*n* = 76) - Department of Addiction Medicine, Hospital for Psychiatry, Psychotherapy, Psychosomatic Medicine, Bayreuth, Germany (*n* = 24)	- Department of Oral and Maxillofacial Surgery, Klinikum rechts der Isar, Munich, Germany (*n* = 66) - Dental surgery clinic, Augsburg, Germany (*n* = 34)
Demographic characteristics	- Mean age: 29.4 years (SD ± 7.4) - Gender distribution: 83 males, 17 females	- Mean age: 29.3 years (SD ± 7.2 years) - Gender distribution: 83 males, 17 females
Mean duration of MA use	- 6.9 years (SD ± 4.6)	- None

*MA* methamphetamine, *SD* standard deviation

For the comparison group, we defined the matched-pair criteria gender and age (+/−1a). Participants for the matched-pair group were selected from hospitalized patients at the University Hospital of the Munich University of Technology and from patients of an ambulatory dental surgery in Augsburg, Germany. Hospitalized patients at the University Hospital who had good general health and no clinical abnormalities and who planned a short in-patient stay of no longer than 1 day postoperatively were assessed for possible study participation. Operations planned on these patients were: 1. removal of osteosynthesis plates after small midfacial or jaw fractures; 2. removal of odontogen cysts; 3. surgical extraction of wisdom teeth. Group 1 had undergone a healing process of, on average, 6 months after small fractures with no complications during healing and with no persisting clinical symptoms. Group 2 included patients with radiologically diagnosed cysts with no clinical symptoms. All wisdom teeth of Group 3 were asymptomatic with no signs of inflammation. Therefore, these hospitalized patient groups could be equated with a generally healthy study population. The examination of these patients was always performed before the planned operation. Patients at the ambulatory dental surgery with good general health without intraoral wounds, feelings of pain, infections or recently performed invasive therapies were assessed for possible study participation. The scheme of examination was the same in both comparison groups.

### Data collection

**Fig. 1:**
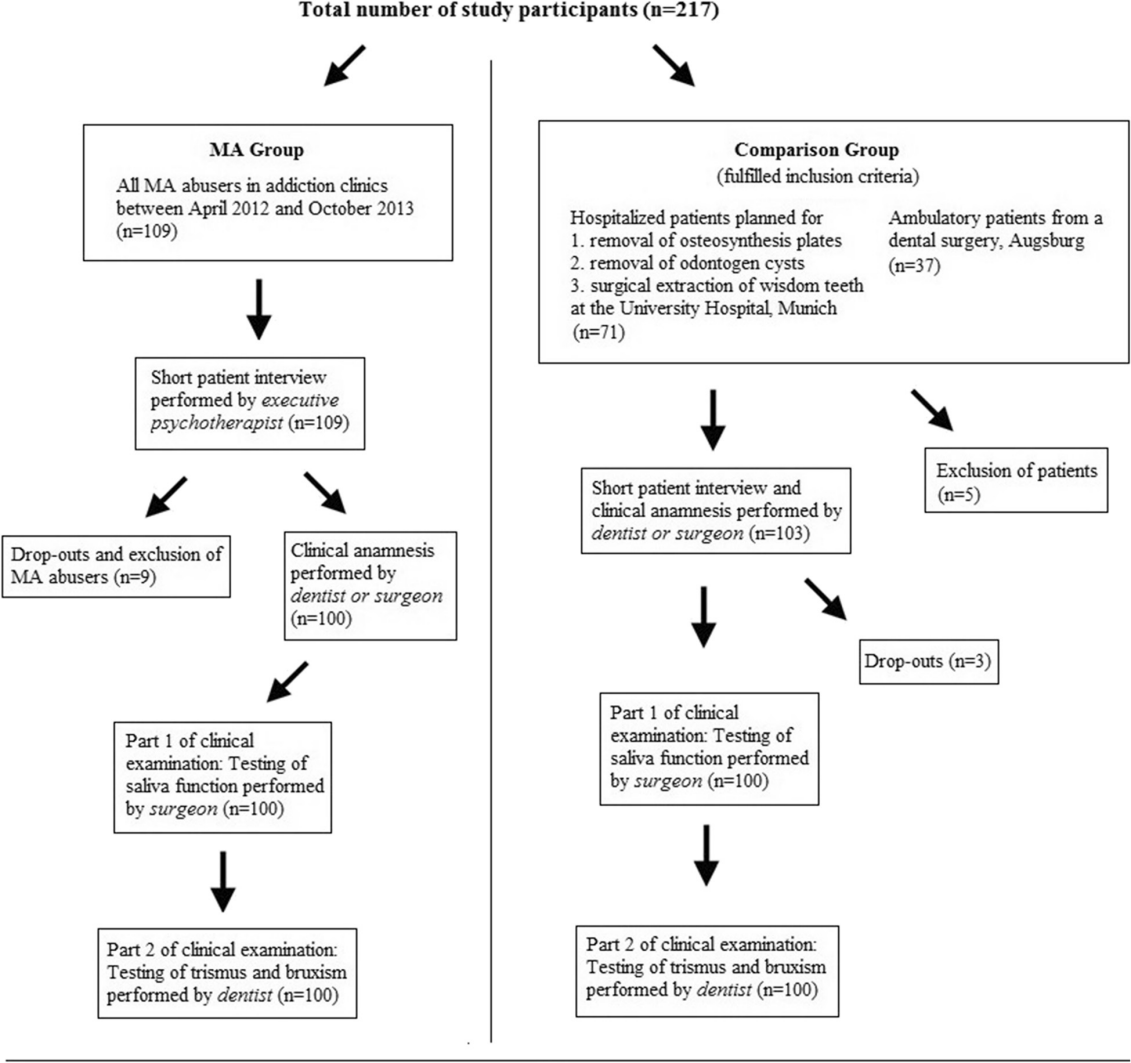
Examination scheme of all study participants

Before the start of the clinical examination, every MA abuser was asked, in a short clinical anamnesis, whether he or she noticed following specific symptoms from the beginning of MA abuse: 1. dry mouth; 2. jaw clenching; 3. pain in the temporomandibular joint (TMJ). If so, the clinical symptoms were noted on the patient’s individual examination form. The short clinical anamnesis was performed by either the dentist or the maxillofacial surgeon. As in the MA group, a short patient interview was performed in the control group by either the dentist or the surgeon. The structure of the interview was almost the same as that of the MA group. First, the inclusion criteria were examined (matched-pair criteria gender and age). Subsequently, if the inclusion criteria were fulfilled, the exclusion criteria were checked (any use of addictive substances). If the potential study participant did not fulfil the exclusion criteria, the information document was handed out combined with an informative conversation with the either the dentist or the surgeon, if desired. If the patient gave written consent, the clinical examination followed. 97 % of all questioned hospitalized and ambulatory patients agreed to participate in the study. The examination appointments of the comparison group took place subsequent to and promptly after each examination appointment of the MA group. The complete examination scheme is shown in Fig. 1.

In the first section of the clinical inquiry, the test persons were initially screened for evidence of potential dry mouth from chronic MA consumption. For this purpose, measurements of the saliva flow rates and total saliva production in milliliters within 5 min were taken by using the sialometry method. Each test person was administered a saliva-stimulating small paraffin wax cube (CRT Paraffin, Vivadent Company, Ellwangen, Germany) and requested to chew on it for 30 s. The test persons were then asked to spit out the soft wax cubes and to swallow all the saliva accumulated in the mouth. From that time on, over a period of five minutes, the saliva produced by the test persons was collected in a sterile graduated measuring receptacle and immediately drawn up into a 5 ml syringe and measured. Subsequently, the total amount of saliva was noted. Subsequently, the individual saliva buffering capacities were tested by means of saliva samples. We used the CRT® buffer test (Vivadent Company, Ellwangen, Germany), the test principle of which included a determination of the pH-value of the saliva. A pH-indicator was located on acid-treated test sticks. A saliva sample from each participant was pipetted onto a pH-indicator stick. If the saliva could buffer the acids on this test stick, the colour of the indicator stick changed. The grading of the buffer capacity was indicated by five different degrees of change of colour of the indicator surface after a response time of exactly 5 min. The classification of the five different degrees was carried out in accordance with the manufacture’s specifications of the test and as reported by previous authors [17]. The complete first section of the clinical examination with respect to saliva function was performed by the maxillofacial surgeon in all study participants.

In the second section of the clinical inquiry, we evaluated potential trismus and bruxism in connection with chronic MA consumption. In order to assess potential trismus, the patient was asked to open his/her mouth at maximum angle. The cutting edge distance of the median upper jaw and lower jaw incisor were then marked on a wooden spatula, measured up with a ruler and noted as “maximum opening of the mouth” (MOM). For evaluation of potential bruxism, the following criteria were checked: 1. tooth attrition in more than 50 % of all teeth, 2. dentine exposure through at least three occlusal surfaces; 3. macroscopic visual enamel cracks in at least three teeth. If a participant fulfilled at least one of these three criteria, he or she was assessed as being positive for bruxism. The complete second section of the clinical examination was performed by the dentist in all study participants.

### Statistical methods

We used the software programs SPSS 23.0. (IBM, Armonk, USA) and Cytel Studio version 10 (Cytel, Cambridge, USA) for the statistical analysis and Microsoft Excel 2010 (Microsoft, Redmond, USA) for data transfer and handling. The complete data analysis was performed in cooperation with the Institute for Medical Biometry, Epidemiology and Medical Informatics of the Saarland University, Germany. For each parameter, an adequate statistical test was selected. *p*-values were subject to a significance level of 0.05. For MOM and total saliva production, we used the t-test for dependent samples by comparing means. For saliva buffer capacity, we used the Wilcoxon signed-rank test for comparing medians. For bruxism, we employed Mc Nemar’s test. The two-sided level of significance was set at α = 0.05. Multiple testing was accounted for by applying the procedure of Bonferroni-Holm, i.e. by sorting *p*-values from the lowest to the highest and comparison with the local adjusted significance level α/i, where i is the order of the respective *p*-value p_i_.

## Results

**Fig. 2:**
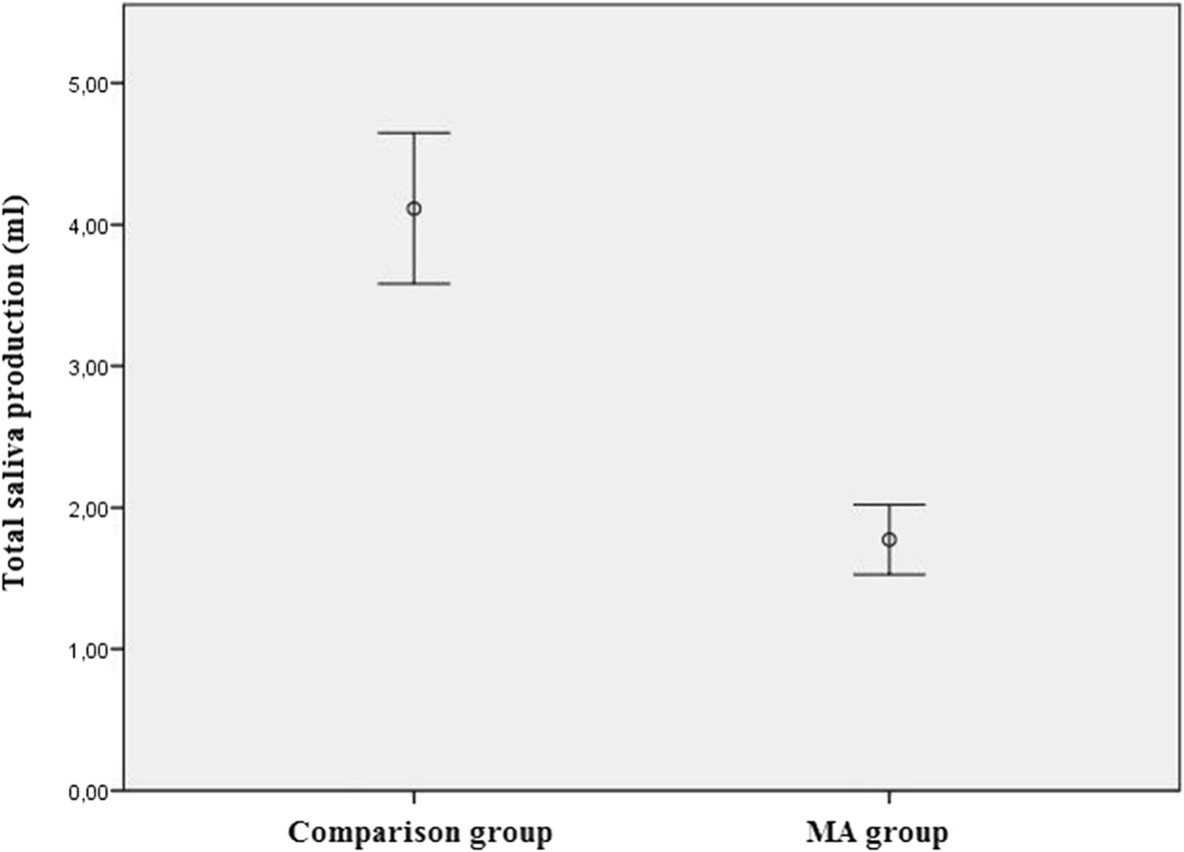
Mean stimulated total saliva production within 5 min in both groups (*p* < 0.001)

The majority of methamphetamine abusers reported a dry mouth (72 %) and jaw clenching (68 %) from the beginning of MA abuse. Almost half of all MA abusers noticed pain in the TMJ (47 %). With respect to the clinical examination, the total quantity of saliva was significantly lower in the MA group in contrast to the comparison group (*p* < 0.001). The median total quantity of saliva of the MA group here was 1.8 ml (SD ± 1.2), whereas in the comparison group, the average volume was 4.1 ml (SD ± 2.7) of total saliva (Fig. 2). The observed power calculation for the results of “total saliva production” showed a 99 % power to detect a difference in means of −2.3 by using a two group Satterthwaite t-test with a 0.05 two-sided significance level. Among the MA group, the buffer capacity (BC) of saliva was also significantly lower (*p* < 0.001). Only 9 % had high BC (pH above 6), 11 % medium-high (pH from 6–5.5) and 43 % medium BC (pH from 5.5–4.5); 30 % of MA consumers displayed low-medium (pH from 4.5–4) and 7 % low BC (pH below 4). Among the comparison group, 55 % revealed high, 25 % medium-high and 18 % medium BC. Merely 3 % had low-medium BC; low BC was not apparent among the comparison group. In the MA group, the MOM averaged 48.7 mm (SD ± 6.6), whereas in the comparison group, it was 48.1 mm (SD ± 6.2). A statistically relevant difference could not be discerned (*p* = 0.494). Bruxism criteria were fulfilled among 81 % of MA consumers, whereas in the comparison group, this percentage was only 39 %. Thus, significant differences exist between the two groups (*p* < 0.001). All clinical data are shown in Table 2.

**Table 2:** Clinical data

		MA group (*n* = 100)	Comparison group (*n* = 100)	*p*-value
Trismus and bruxism				
Maximum mouth opening (mm)	Mean (SD)	48.7 (6.6)	48.2 (27.2)	0.494
Bruxism symptoms (*n* = 100)		81	39	<0.001
Saliva flow rate				
Total saliva production (ml/5 min)	Mean (SD)	1.8 (1.2)	4.1 (2.7)	<0.001
Saliva buffer capacity	%			<0.001
High		9	54	
High-medium		11	24	
Medium		43	18	
Medium-low		30	3	
Low		7	0	

*MA* methamphetamine, *SD* standard deviation

## Discussion

The results of the study indicate that sympathomimetic effects of the substance MA contribute to the highly damaging potential on the stomatognathic system in cases of chronic MA abuse. We have found a significantly lower saliva rate and significantly higher signs for bruxism within the MA group in comparison with a same-aged and same-gender group with no history of MA abuse. Furthermore, chronic MA abuse reveals a detrimental influence on saliva buffer capacity.

Many devastating effects of MA on oral health resulting in the term “meth mouth” are described in the current literature [1, 8, 10, 13]. Dry mouth or even xerostomia is one of the main symptom described in scientific publications as occurring with chronic MA abuse [11, 16] and with respect to “meth mouth” syndromes [5, 8–10]. The mean standard stimulated salivation is stated at 1–2 ml/min, with salivation of >0.7 ml/min being considered rather low [18]. In this study, the flow of saliva averaged only 0.36 ml/min among 100 chronic MA consumers. Furthermore, the majority of all MA abusers experienced a dry mouth from the beginning of MA abuse. Thus, we can confirm the symptom of a dry mouth and the high risk potential of xerostomia in cases of chronic MA abuse. Xerostomia is accompanied by the loss of important protective features of saliva. Correspondingly, the lack of salivation correlates with increased caries incidence, gingivitis or periodontitis [19]. To a great extent, the reasons for a dry mouth or even xerostomia induced by MA are still unknown. However, the sympathomimetic essential effect of methamphetamine on the alpha-2-receptors of the brain is the main reason for this [16]. The direct stimulation of the inhibitory alpha-2-adrenal receptors of the salivary glands by MA as a possible mechanism is, nevertheless, considered to be controversial [11, 16]. In our study, we have not evaluated the pathogenic mechanism of the symptom of dry mouth but we consider that its development is a composite of the factors described above. We have confirmed previous assumptions of a high risk for xerostomia and subsequently caries decay in MA abusers. These previous assumptions were primarily based on case reports [5, 13]. We have detected only one systematic study involving the examination of salivary flow rate and buffer capacity with 28 MA abusers and 16 comparison subjects; tooth wear was noted but no significant decrease in saliva flow rates among the MA abusers [20]. This study includes a small sample size, possibly explaining the results being divergent from those of our study. We have found a significant decline of saliva production in the MA group; this is valuable information for the development of specific preventive and therapeutic strategies in order to prevent a probable “meth mouth” syndrome in MA abusers.

Saliva also plays an important part in the prevention of erosive lesions [21, 22]. The erosion of enamel is facilitated when saliva flow rates and buffer capacity are lowered [21]. Under MA-influence, in particular, the pH of saliva drops and the diminution of the buffer capacity has been noticed [20, 23]. Navarro et al. examined the effects of MA on saliva pH on the basis of eight test persons who took a placebo or an MA derivative orally according to a double-blind randomized study design [23]. In a comparison of the two groups after four hours, the pH dropped by 0.6 in the group that had taken the MA derivatives [23]. Ravenel et al. also compared saliva pH and saliva buffer capacity between 28 MA consumers and 16 healthy comparison test persons and found a lower pH and lower saliva buffer capacities among the MA group [20]. The results of our study also show a significant lower saliva buffer capacity on MA consumption. Together with the results of previous findings, these data allow the conclusion to be made that saliva pH drops and saliva buffer capacity is reduced with chronic MA abuse. The underlying mechanism of action of MA on the pH-value in the saliva and on the buffer capacity has not been clarified adequately as yet and has not been evaluated in this study. Presumably, reduced salivation and its reasons are decisive factors, as maximum saliva buffer capacity is essentially achieved by salivation [23].

Excessive neuromuscular activity among MA consumers can cause parafunctional temporomandibular function accompanied by intensified bruxism [24]. MA consumers tend to clench and grind their teeth strongly [8]. Trismus also frequently occurs because of excessive neuromuscular activity on chronic MA consumption [10]. Thus, parodontal diseases and temporomandibular dysfunctions can be the consequences of permanent bruxism and trismus [8]. In order to determine potential trismus and bruxism, attention has been paid to potential painful restrictions in the opening of the mouth on the basis of measurements of MOM and to the excessive numbers of dental cut facets with possibly even dentine exposure or enamel cracks. However, among the comparison group, the results of the maximum opening of the mouth displays only slightly larger openings of the mouth than among the MA group. This might be explained by the intensified neuromuscular activity causing strong grinding, tenseness and trismus during acute phases of consumption [8], whereas in phases of no consumption, the “recovery” of masticatory muscles and, thus, the reversibility of jawjoint symptoms might occur. However, bruxism symptoms could be found in a large number of patients (81 %) with a significant difference in comparison with the group without MA abuse (*p* > 0.001). Moreover, the majority of MA abusers reported jaw clenching and almost half of all abusers indicated pain in the TMJ from the beginning of MA abuse. These findings reveal a high risk for enamel loss and tooth wear in cases of MA use like and are in line with previous descriptions. In particular, the combination of bruxism and reduced saliva buffer capacity, as also observed in the MA group, might trigger the risk for dental erosions and enamel loss significantly.

The findings of our study need to be viewed in context with few limitations. Our evaluation of extensive grinding facets in the bruxism part and the colour assessment in the buffer capacity part of the study depends on the examiner’s subjectivity. Furthermore, we had to rely on the truthful answers of all study participants with regard to inclusion and exclusion criteria. Despite these limitations, the study concept and its findings show several strengths including a systematic clinical evaluation in a study population that is usually difficult to reach and for which systematic study designs rarely exist. The large sample size of this study provides high significance of the examined parameters. Furthermore, the simultaneous examination of a matched-pair comparison group guarantees optimal comparability of data and verifies methods of data collection. In the future, additional research involving systematic clinical intraoral examinations, preferably with standardized indices and a longitudinal study design, is recommended to confirm the findings of this study and to gain a clearer view of the “meth mouth” phenomenon.

## Conclusion

In summary, the sympathomimetic central stimulant MA has a strong impact on the stomatognathic system. The findings of this study reveal significantly lower saliva production in MA users; this might result in dry mouth or even xerostomia in the long-term and, consequently, might increase the risk for oral diseases such as carious lesions, oral tissue infections or erosions. Sympathomimetic effects of MA lead to extensive jaw clenching with tooth wear and loss of enamel. Additionally, MA abusers show a worse saliva buffer capacity significantly, whereby dental erosion will be favored. Based on the aforementioned findings, the authors recommend specific preventive and therapeutic strategies in order to avoid potential “meth mouth” syndrome and to support the social rehabilitation of MA abusers.

## Abbreviations

BC, buffer capacity; CM, Crystal meth; MA, methamphetamine; MOM, maximum opening of the mouth; TMJ, temporomandibular joint.
